# Optimization of nutrient media for sweetpotato ( *Ipomoea batatas L.*) vine multiplication in sandponics: Unlocking the adoption and utilization of improved varieties

**DOI:** 10.12688/gatesopenres.12879.1

**Published:** 2018-11-19

**Authors:** Phabian Makokha, Lexa G. Matasyoh, Reuben T. Ssali, Oliver K. Kiplagat, Bramwel W. Wanjala, Jan Low

**Affiliations:** 1 International Potato Center, Nairobi, Sub-Saharan Africa, Kenya; 2 Department of Biological Sciences, University of Eldoret, Eldoret, Kenya; 3 International Potato Center, Kampala, Sub-Saharan Africa, Uganda; 4 Department of Biotechnology, University of Eldoret, Eldoret, Kenya

**Keywords:** Sandponics, optimizing, sweetpotato vines, pre-basic seed

## Abstract

**Background:** Sweetpotato, being a vegetatively propagated crop is prone to seed degeneration, and a continuous source for high quality sweetpotato seed is critical for an efficient seed system.  In most Sub-Saharan African countries, the National Agricultural Research Systems use tissue culture to produce limited quantity of pre-basic sweetpotato seed which is then used as starting material to maintain and produce basic seed in mini-screen houses, net tunnels or open field multiplication in low-virus pressure areas by either the private seed companies or vine multipliers. Soil is the predominant media for pre-basic seed multiplication. Multiplying pre-basic sweetpotato seed in sand with fertigation, also known as ‘sandponics’ is a possible opportunity towards sustainable production of pre-basic sweetpotato seed. It would be beneficial to examine the feasibility and the potential to replace soil system with ‘sandponics’ for growing pre-basic sweetpotato seed.

**Methods:** Pot experiments were conducted to study how sweetpotato vine propagation is affected by sequentially omitting nitrogen, phosphorus, calcium, sulfur and boron from fertilizer applications on cv. Kabode. The experiment was laid in a randomized complete block design with five levels of the factor fertilizer, replicated four times with two blocks. The effect of fertilization of nitrogen at (0, 100, 150, 200 & 250), phosphorus at (0, 30, 60, 90 & 120), calcium at (0, 100, 200, 300 & 400), sulfur at (0, 30, 60, 90 & 120) and boron at (0, 0.1, 0.2, 0.3 & 0.4) ppm on sweetpotato vegetative growth parameters was measured 45 days after planting.

**Results:** The obtained results showed that application of 200, 60, 200, 120 and 0.3 ppm of N, P, Ca, S and B respectively recorded the highest values in sweetpotato vegetative growth parameters.

**Conclusions: **These results imply that pre-basic sweetpotato vine yields in sandponics could be increased by using this optimized media.

## Introduction

Sweetpotato ( *Ipomea batatas L.*) is a staple food for smallholder farmers in much of Sub-Saharan Africa. The crop has an annual worldwide production of more than 105 million metric tons ( FAOSTAT, 2016). In Kenya, sweetpotato is an important crop grown widely by small scale farmers, mainly women, and plays an important role, both as a source of household food security, and a source of family cash income ( Carey *et al*., 1997). Due to its nutritional qualities (rich in carbohydrates, dietary fibre, beta carotene, vitamin C and vitamin B6), sweetpotato is considered as a crop with great potential for human consumption ( Bovell-Benjamin, 2007). The crop is in nearly all agro-ecological zones of Kenya with higher concentration in the lower midland zones ( Ndolo *et al*., 2001). The national average farm yield of sweetpotato in Kenya is only 14.7 t/ha ( FAOSTAT, 2016) compared to 50 t/ha observed under experimental conditions. The wide yield gap is caused by several factors including wide spread use of low yielding traditional varieties, pests, poor cultural and post-harvest practices, inadequate storage facilities, lack of drought tolerant varieties and poor-quality planting materials.

Production of sweetpotato is still constrained by lack of access to quality planting material at the right time and in the right quantities. This is mainly due to infection of planting materials with the sweetpotato virus disease (SPVD) and prolonged droughts that either compromises the quality or availability of vines from the previous season’s crop. Insufficient planting materials limit the adoption and utilization of improved sweetpotato varieties ( Namanda, 2012). Multiplication of pre-basic sweetpotato vines in screenhouses is possible in either sterilized soil or sand. However sterilizing soil with either diesel or firewood translates to very high running costs and requires a lot of labor while using sand with an irrigation system, also known as ‘sandponics’, may require a high initial investment that necessitates high vine yields for the multiplier to break even. This study aimed to determine the conditions for high vine yields in sandponics system by optimizing the nutrient media to maximize vegetative growth.

## Methods

A screenhouse experiment was conducted between March and May 2018 at the Kenya Plant Health Inspectorate Services (KEPHIS), Muguga, Kenya, located at 1° 11’ 0” South, 36° 39’ 0” East at an altitude of about 1950m above sea level. The study involved sequentially increasing levels of nitrogen, phosphorus, sulfur, calcium and boron on sweetpotato vine yields in sandponics ( Table 1). The first level was by omitting the nutrient under study, this involved substituting sources for some of the nutrients. A sample of irrigation water was collected and submitted to Crop Nutrition Laboratories, Kenya for nutrient analysis using colorimetric ( Oser & Summerson, 1947) and spectroscopy ( Fishman & Downs, (1966)) methods prior to experiment set off and the report was used to adjust final nutrient concentrations for the studied elements.

**Table 1.** Rates of nutrient application in parts per million (ppm).

Element	Concentration levels (ppm)
Nitrogen	0, 100, 150, 200, 250
Phosphorus	0, 30, 60, 90, 120
Calcium	0, 100, 200, 300, 400
Sulfur	0, 30, 60, 90, 120
Boron	0, 0.1, 0.2, 0.3, 0.4

All the fertilizers used in the study ( Table 2) easily dissolved in water apart from calcium triple superphosphate. Weighed fertilizers were dissolved separately in approximately one litre of water until they were completely dissolved. Calcium triple superphosphate granules were placed in a 50-mesh insect-proof net from Amiran Kenya and soaked in a bucket of warm water overnight and the following day were squeezed until all granules disappeared. The solution was left to settle down and clear supernatant transferred discarding impurities at the bottom. The dissolved fertilizers were transferred into black buckets and the volume topped up to 50 L with water, finally the pH was adjusted to 5.8 using HI98107 pH meter (Hanna Instruments Ltd, UK) by adding 3 mL of 0.1 M phosphoric acid.

**Table 2.** Sources of individual target elements used in the experiment.

Fertilizer compound	Composition	Supplier
Calcium Nitrate (Calcinit)	15.5%N, 19%Ca	Amiran Kenya
Calcium Triple Phosphate	46%P _2_O _5_	Amiran Kenya
Magnesium Sulfate	26%S	Amiran Kenya
Microsol B	2.5%B, 0.036%Mo	Amiran Kenya
Calcium Chloride	27.9%Ca	Amiran Kenya
Magnesium Nitrate	10%N 13%Mg	Amiran Kenya

Heat controllers, sensors and cooling air extract fans were installed in the screenhouse to maintain a temperature range of 26±4 ^o^C ( Jiang, 2013) which is optimal for sweetpotato vine growth and this was monitored by HOBO U12-013 data logger (Onset Computer Corp., Bourne, MA, USA). Sieved river sand was soaked for 10 minutes in 10% sodium hypochlorite bleach (NaOCl), then rinsed three times with clean water to remove any excess bleach.

### Experimental design and tested treatments

The experiment was laid in a factorial arrangement in a randomized complete block design with five levels of the factor fertilizer, replicated four times with two blocks. Pathogen tested plantlets of cultivar ‘Kabode’ obtained from KEPHIS were weaned and multiplied in the greenhouse to generate experimental planting materials. Ten cuttings of three nodes were planted in a plastic pot measuring 18 cm diameter and a slanting 20 cm height filled with 5.5 kg of sterilized river sand.

### Management practices

Plants were manually fertigated using graduated measuring jars at the base of the sweetpotato plants until all the pots started to leak the nutrient solution to the plate at the bottom of the pots. An Irrometer SR 12ʹʹ (Irrometer Company Inc., CA, USA) was used to schedule fertigation frequency.

### Recorded data

Data on petiole length, internode length, vine length, vine girth, leaf area and nodes produced were recorded 45 days after planting (Dataset 001 ( Makokha, 2018)). Petiole length, internode length and vine length were measured using a meter ruler.

**Petiole length (cm);** this was determined by measuring the point between leaf attachment to the main stem and the leaf, the measurements were done on the fifth leaf from the tip of the main stem.

**Vine length (cm);** this was determined by measuring the main stem length from the surface of the soil in the pot to the tip.

**Internode length (cm);** this was determined by measuring the fifth internode from the tip of the main stem.

**Leaf area (cm ^2^);** this was determined by measuring leaf length (L) and width (W) at the widest part of the 5 ^th^ leaf from the tip of the main stem and the product L × W was used to compute for leaf area (cm ^2^/plant).

**Vine girth (cm);** this was determined by measuring the fifth internode girth diameter from the tip of the main stem using vernier caliper.

**Nodes produced;** these were counted on the main stem from the surface of the pot to the fully open leaf at the tip of the vine.

**Nutrient disorders;** nutrient disorders were visually observed and recorded.

**Shoot fresh and dry mass (g);** at harvesting, four vines from each replication for each treatment were sampled to constitute a composite sample for above ground biomass determination. Harvested fresh shoot samples were separated from roots for each treatment, weighed and then heated to a constant weight in an oven for 48h at 65°C. These were re-weighed to determine the dry weight (Dataset 002 ( Makokha, 2018)).

**Fresh and dry root mass (g);** composite samples for fresh root samples from four plants for each replication from each treatment were harvested, weighed and heated to a constant weight in an oven for 48h at 65°C and these were then reweighed to determine the dry weight (Dataset 003 ( Makokha, 2018)).

**Leaf tissue analysis;** the blades of the 7 ^th^ to 9 ^th^ youngest leaves from the shoot tip were randomly selected as the index tissue to form a composite sample ( O’Sullivan *et al*., 1996b) and taken to Crop Nutrition Laboratories, Kenya for tissue analysis. The nutrient values in the leaf samples were plotted against various measured growth parameters and results used to extrapolate the optimal nutrient concentrations for sweetpotato vine growth for the 5 elements ( Figure 2).

### Statistical analysis

The collected data on growth parameters was subjected to analysis of variance using SAS 9.4 version ( SAS institute, 2001) and means were separated using Dunnett’s test of least significance difference at 0.05 level of probability.

## Results and discussion

### Nitrogen rate

**Figure 1.**
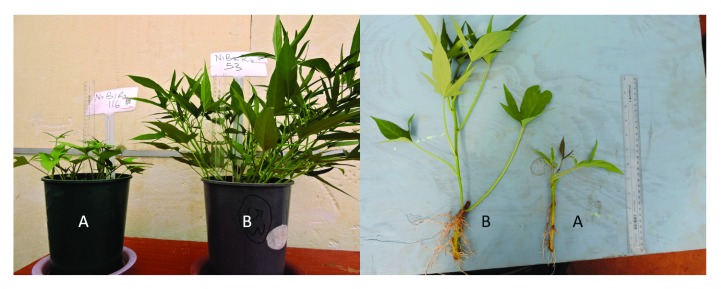
( **A**) Photograph of N deficient vines on the minus N treatment against the all ( **B**) nutrient control 30 days after planting.

Plants in the N omitted (0 mg NL ^-1^) pots exhibited N deficiency symptoms two weeks after planting when the crop had two fully expanded leaves. Plants showed stunting with minimal expansion of the leaf area ( Figure 1A), as compared to plants on receiving all nutrient ( Figure 1B). There was reddening of basal leaf edges advancing to younger growing leaves.

**Figure 2.**
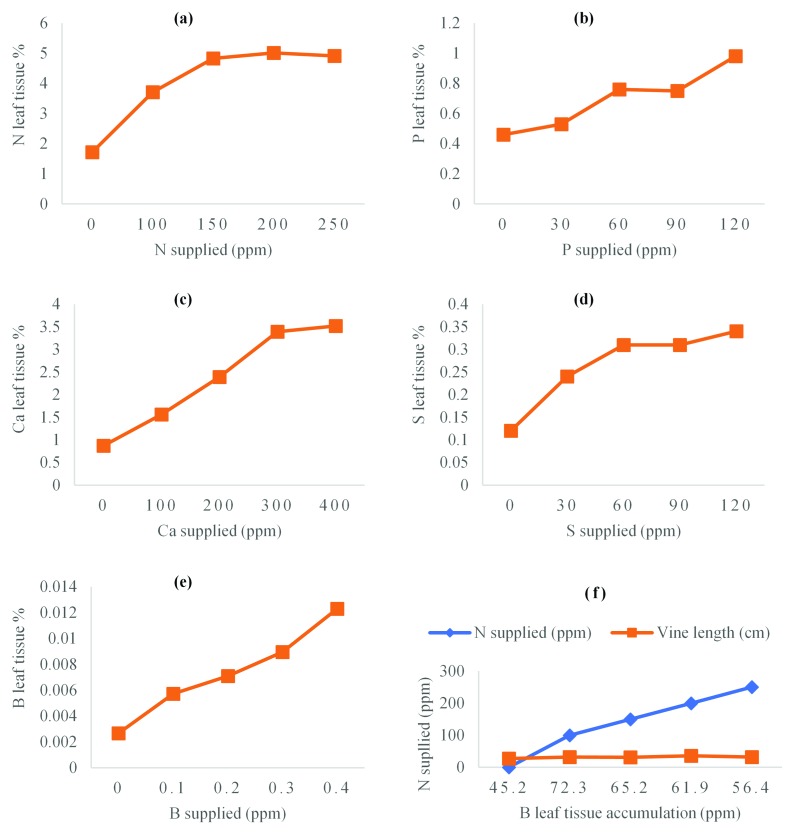
Concentrations of ( **a**) N, ( **b**) P, ( **c**) Ca, ( **d**) S and ( **e**) B in the index tissue across a range of supply levels. Critical concentrations were extrapolated using an ( **a**) exponential function model, ( **b**) regression and ( **c**) the ‘broken stick’ procedure. Figure 2 ( **f**) response of vine length to nitrogen (N) boron (B) interaction.

Results from the experimental ( Table 3) study showed that the means of internode length, leaf area, petiole length, vine girth, vine length and nodes production were significantly affected by increasing N rates ( Table 4). When 0 ppm and 100 ppm N were applied internode length, leaf area, petiole length, vine length and nodes produced were significantly lower compared to 150, 200 and 250 ppm N application. The tissue N level recorded was below 4.0% N at 0 and 100 ppm treatments ( Figure 2 (a)).

**Table 3.** Vegetative growth parameters of sweetpotato plants as affected by nitrogen at different rates.

N (ppm)	IL (cm)	LA (cm ^2^)	PL (cm)	VG (cm)	VL (cm)	Nodes	N leaf tissue (%)	AGFW (g)
0	1.2 ^c^	19.7 ^b^	6.9 ^b^	0.7 ^b^	8.1 ^c^	1.3 ^c^	1.72	3.76
100	2.1 ^ba^	39.5 ^a^	10.7 ^a^	0.9 ^a^	27.4 ^b^	3.6 ^b^	3.71	16.1
150	2.2 ^a^	46.8 ^a^	12.6 ^a^	1.0 ^a^	33.3 ^a ^	4.3 ^ba^	4.83	23.1
200	2.0 ^ba^	49.7 ^a^	12.0 ^a^	0.9 ^a^	31.7 ^ba^	4.1 ^ba^	5.01	20.2
250	1.7 ^b^	48.9 ^a^	11.4 ^a^	0.9 ^a^	30.6 ^ba^	4.4 ^a^	4.91	21.4
LSD (5%)								

*Means followed by the same letter within a column are not significantly different at 5% according to Dunnett’s test; N = nitrogen; IL = internode length; LA = leaf area; PL = petiole length; VG = vine girth; VL = vine length; AGFW = above ground fresh weight; Nodes = number of 3 node cutting produced per vine.*

**Table 4.** Means of sweetpotato vegetative growth parameters as influenced by increased N, P, Ca, S and B fertilization.

N (ppm)	IL (cm)	LA (cm ^2^)	PL (cm)	VG (cm)	VL (cm)	Nodes
0 100 150 200 250	1.2 ** 2.1 ** 2.2 ** 2.0 * 1.7	19.7 ** 39.5 * 46.8 49.7 48.9	6.9 ** 10.7 12.6 11.9 11.3	0.7 ** 0.9 1.0 0.9 0.9	8.1 ** 27.4 33.3 31.7 30.6	1.3 ** 3.6 ** 4.3 4.1 4.4
Mean LSD (5%) CV	1.8 0.4 15.0	40.9 12.3 20.9	10.7 2.3 15.3	0.9 0.06 4.9	26.2 5.0 13.4	3.5 0.8 15.2
P (ppm)	IL (cm)	LA (cm ^2^)	PL (cm)	VG (cm)	VL (cm)	Nodes
0 30 60 90 120	2.1 2.2 2.2 2.0 2.1	47.2 50.0 53.4 52.6 51.7	12.3 14.0 12.7 13.5 13.2	0.9 0.9 0.9 0.9 0.9	31.2 31.8 33.8 34.2 31.5	3.9 3.6 4.0 3.8 3.4
Mean LSD (5%) CV	2.1 0.5 16.7	51.0 13.5 18.4	13.2 4.0 21.1	0.9 8.5	32.5 9.2	3.7 13.1
Ca (ppm)	IL (cm)	LA (cm ^2^)	PL (cm)	VG (cm)	VL (cm)	Nodes
0 100 200 300 400	1.7 * 2.0 2.4 2.3 2.1	35.8 * 46.1 47.1 44.8 45.5	9.3 12.4 11.8 11.9 11.2	0.9 0.9 0.9 0.9 1.0	27 * 37 36.4 33.5 33.6	3.7 4.5 4.2 4.4 4.2
Mean LSD (5%) CV	2.1 0.4 15.6	43.9 9.6 17.2	11.3 2.8 19.6	0.9 0.05 4.9	33.6 0.05 13.2	4.2 0.7 14.1
S (ppm)	IL (cm)	LA (cm ^2^)	PL (cm)	VG (cm)	VL (cm)	Nodes
0 30 60 90 120	2.2 * 2.1 ** 2.3 ^ns^ 2.1 ** 2.5	39.6 44.1 51.0 45.7 43.8	12.5 ^ns^ 12.2 * 13.2 11.3 14.3 **	0.9 1.0 0.9 0.9 0.9	32.3 36.1 35.5 31.4 35.5	4.1 4.5 4.4 4.1 4.3
Mean LSD (5%) CV	2.2 0.4 12.0	44.8 10.7 16.6	12.7 2.8 15.5	0.9 0.1 6.5	34.2 6.0 12.2	4.3 1.1 18.7
B (ppm)	IL (cm)	LA (cm ^2^)	PL (cm)	VG (cm)	VL (cm)	Nodes
0 0.1 0.2 0.3 0.4	1.8 2.1 2.3 2.2 2.1	40.4 43.0 48.1 49.8 46.9	9.8 12.2 12.4 11.4 10.9	0.9 0.9 0.9 1.0 0.9	27.7 * 31.8 31.0 36.2 32.6	3.9 4.0 4.0 4.3 4.0
Mean LSD (5%) CV	2.1 0.5 16.5	45.6 9.5 14.5	11.3 3.0 18.2	0.9 0.08 6.2	31.8 6.3 13.7	4.0 0.9 16.3

*, ** significant at 0.05 and 0.01 respectively N = nitrogen; P = phosphorus; Ca = calcium; S = sulfur; B = boron; IL = internode length; LA = leaf area; PL = petiole length; VG = vine girth; VL = vine length; Nodes = number of 3 node cuttings per vine; B = Boron

The highest N rate (250 ppm) applied resulted in reduction but not significant growth of internode length, leaf area, petiole and vine length compared to plants grown at 200 ppm N ( Table 3). Nitrogen levels in the tissue increased logarithmically as fertilizer rate increased up to 200 ppm N fertilization ( Figure 2 (a)).

**Figure 3.**
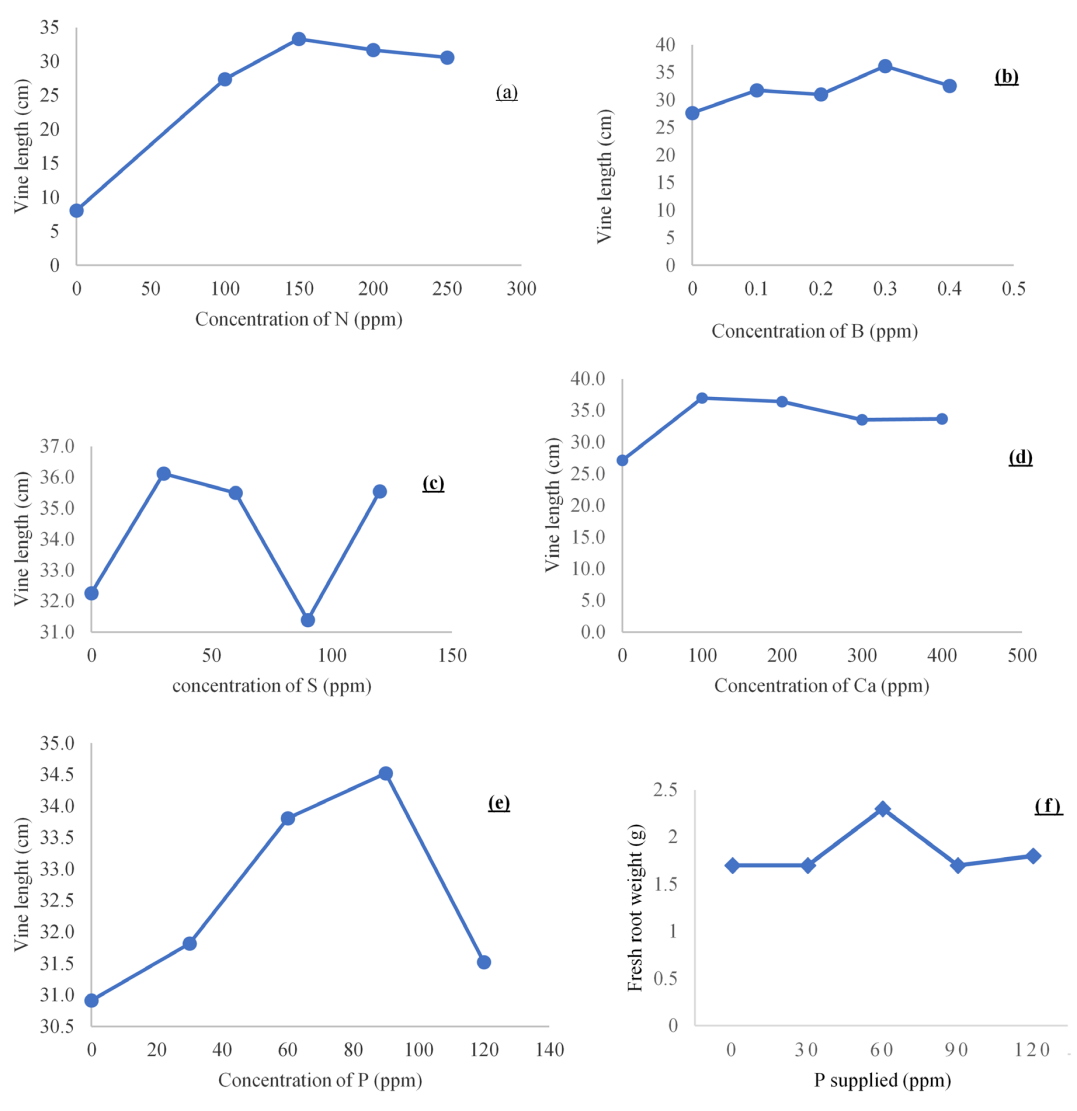
Relationships between sweetpotato vine length and the supply of ( **a**) N, ( **b**) B, ( **c**) S, ( **d**) Ca and ( **e**) P across a range of levels and ( **f**) relationships between fresh root weight and the concentrations of P supplied.

Results in the growth of internode length, leaf area, petiole length, nodes production, above ground fresh weight and N tissue accumulation suggest that optimal growth in these parameters can be achieved when N concentration is 200 ppm. This data showed that as N rate increased from 0 – 250 ppm, there was an increased significant growth in the vine length, vine internode length, leaf area, petiole length and nodes production. Increase in above ground fresh biomass accumulation was also noted ( Table 3), however, a decline in the growth increase in vine length was observed as N rate exceeded 200 ppm ( Figure 3 (a)). The leaf tissue analysis report indicated ( Figure 2 (a)) that as N fertilization increased, N level in the plant tissue increased when its value was relatively lower than 5.01%. After N tissue level neared or exceeded 5.01%, the subsequent value, 4.91 % which is lower indicated that there was no further increment of N levels in the plant tissue. This information indicated that when N tissue was approximately 5.01% or above it might be toxic. This data further showed that significant growth in vine length, internode length, leaf area and petiole length occurred as N fertilization increased until 5.01% N accumulation in plant tissue when no further increase was observed, an indication that 5.01% N tissue was the optimal nutrient concentration for sweetpotato vine growth. The minimal growth in internode length, leaf area, petiole length, vine length, nodes produced, above the ground fresh biomass and N tissue accumulation at 100 ppm fertilization ( Table 3) shows that when tissue N was approximately 4.0%, it might be deficient an indication that this is the critical nutrient concentration for N deficiency. Jiang (2013) reported similar results.

### Boron leaf tissue accumulation as affected by increased nitrogen application

Mulder (1953) reported that increased nitrogen rate antagonizes boron uptake, however, it is documented that boron plays an important role in sweetpotato growth, primarily in root development ( Li *et al*., 2017; Miller & Nielsen, 1970; Nusbaum, 1946; O’Sullivan *et al.*, 1997; Willis, 1943). Comparing this datum and Mulder’s report, boron levels decreased significantly when nitrogen rates neared or exceeded 200 ppm nitrogen fertilization ( Figure 2 (f)). There was a sharp increase in boron tissue concentration from 45.2 to 72.3 ppm at 0 and 100 ppm nitrogen fertilization respectively, after which followed a successive decrease in boron tissue concentrations to 65.2, 61.9 and 56.4 ppm when plants were fertilized with 150, 200 and 250 ppm nitrogen respectively. The concentrations of boron recovered in leaf blades as a percentage for each of the 5 treatment levels of nitrogen were plotted against the growth in vine length, the maximum growth in vine length was recorded at 200 ppm nitrogen fertilization ( Figure 2 (f)).

Taking into account all the factors above, nitrogen fertilization at 200 ppm seemed to be the most favorable for vine growth. These findings are supported by previous nitrogen studies conducted in greenhouse studies in other crops such as Texas mountain laurel ( *Sophora secundiflora*), Anthurium ( *Anthurium andraeanum Lind.*) and bell pepper ( *Capsicum annum L.*), which all reported a similar range of nitrogen fertilization rate for optimal haulm growth ( Bar-Tal *et al*., 2001; Chang *et al*., 2012; Niu *et al*., 2011). A study conducted by Jiang (2013) on nitrogen fertilization rates and application timings on greenhouse sweetpotato production gave similar results.

It has been reported that growth reduction usually happens when a plant nutrient reaches a toxic range ( Smith, 1962). In this study when nitrogen tissue neared or exceeded 5.01% growth was retarded, and there was a reduction in the growth of plants ( Table 3). This indicated that above 5.01% nitrogen tissue might be the indicator of plant toxicity. A sufficient range for solution cultured sweetpotato 28 days from planting was reported between 4.2 – 5.0% when the 7 ^th^ to 9 ^th^ open leaf blades from the shoot tip were sampled ( O’Sullivan *et al.*, 1997). Mills & Jones (1996) reported a sufficient range for field grown sweetpotato in the middle of the growing season to be between 3.3 – 4.5% when the most recent fully developed leaves were sampled.

Sufficiency ranges for nutrients usually vary considerably with phenological crop stage. Jiang (2013) reported that the highest concentration of nitrogen was found in new leaves, and the nitrogen content became less with the age of the plant. Compared with field grown sweetpotato, leaves obtained from greenhouse shoot production were much younger, and thus should have been expected to have a higher nitrogen content. This case has been documented in other crops. Cucumber plants grown in the greenhouse need a higher range of nitrogen than those in the field, 4.5 – 6.0% versus 4.0 – 5.0% ( Campbell, 2000). Greenhouse lettuce can tolerate an even higher nitrogen level, from 4.5 – 6.5%. Based on this information, the relatively higher nitrogen levels in this study seemed to be reasonable, while the drop in the nitrogen tissue accumulation in the leaf samples was an indicator of optimal concentrations having been reached.

### Phosphorus rate

**Figure 4.**
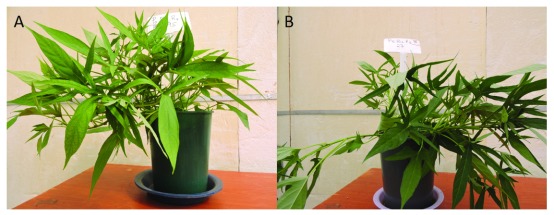
( **A**) Photograph of P deficient vines on the minus P treatment against the all ( **B**) nutrient control 45 days after planting.

Phosphorus fertilization increase did not significantly ( Table 4) affect growth in the means of vine length, leaf area, internode length, petiole length, nodes production and girth size ( Table 5). However, the maximum mean leaf area and internode growth was recorded at 60 ppm phosphorus fertilization. Pronounced visible deficiency symptoms were observed in plants with omitted phosphorus as yellowing of older leaves spreading from discrete interveinal patches affecting half of the blades ( Figure 4 (a)). Healthy vigorous sweetpotato vines were observed on all nutrient control ( Figure 4 (b)).

**Table 5.** Vegetative growth parameters of sweetpotato plants as affected by phosphorus at five rates.

P (ppm)	IL (cm)	LA (cm ^2^)	PL (cm)	VG (cm)	VL (cm)	Nodes	P leaf tissue (%)	AGFW (g)
0	2.1	47.2	12.3	0.9	31.2	3.9	0.46	19.3
30	2.2	50.0	14.1	0.9	31.8	3.6	0.53	20.0
60	2.2	53.4	12.7	0.9	33.8	4.0	0.76	23.7
90	2.0	52.6	13.5	0.9	34.2	3.8	0.75	25.4
120	2.1	51.7	13.2	0.9	31.5	3.4	0.98	23.5
LSD (5%)	ns	ns	ns	ns	ns	ns		

*ns= not significant; IL = internode length; LA = leaf area; PL = petiole length; VG = vine girth; VL= vine length; P = phosphorus; Nodes = number of 3 node cuttings per vine; AGFW = above ground fresh weight.*

There was successive increase in phosphorus levels in the plant tissue as phosphorus fertilization increased ( Figure 2 (b)). The threshold in the increase of P tissue levels was observed at 60 ppm phosphorus fertilization. When phosphorus tissue reached 0.76% a further increase in Phosphorus leaf tissue was not observed as phosphorus fertilization increased, instead there was a non-significant decrease in growth parameters as phosphorus leaf tissue exceeded 60 ppm ( Table 5). Despite the decrease in growth of sweetpotato vegetative growth parameters due to increased P fertilization ( Table 5), the highest growth in vine length was recorded at 90 ppm ( Figure 3 (e)) but was not significant ( Table 4).

Phosphorus fertilization at the 5 levels did not result in significant variation at the 5% confidence level in all growth parameters measured. However, phosphorus deficiency symptoms and decreased non-significant growth of leaf area ( Table 4) and above ground fresh biomass in phosphorus omitted pots was observed, this could have been attributed to the role phosphorus plays in plant physiological processes as reported by Hawkesford *et al.* (2012), Baset Mia (2015) and Siose *et al.* (2017). There is further documented evidence that phosphorus deficiency reduces leaf expansion ( Fageria *et al*., 2003; Fredeen *et al*., 1989; Siose *et al*., 2017), this data confirms these findings. According to Baset Mia (2015), phosphorus deficiency impairs photosynthetic activities in leaf, this is reflected in the low above ground biomass accumulation in this study ( Table 5). The increased growth in leaf area in the phosphorus treated pots could have been a result of the beneficial effect of P on the activation of photosynthesis and metabolic processes of organic compounds in plants which increase the growth of plants ( El Sayed Hameda *et al*., 2011; Purekar *et al*., 1992).

These results further indicated that 30 ppm can be approximated to be the lower critical phosphorus concentration in sweetpotato vine growth, attributed to observed healthy plants. 0.46% phosphorus tissue was recovered from vine leaf blades fertilized at 30 ppm ( Figure 2 (b)), O’Sullivan *et al.* (1997) reported a similar percentage to be within the sufficiency range. The non-significant (P≤0.05) effects of increased phosphorus fertilization on phenological growth parameters could be attributed to increased nutrient imbalance because of sequential phosphorus fertilization ( Kareem, 2013). Olusola (2009) reported that despite higher amount of phosphorus released, recovery of phosphorus fertilizer is about 15 – 30% while about 60% of the phosphorus fertilizer is absorbed. In this study 0.76% phosphorus leaf tissue seemed to be the optimal level for absorption, then further fertilization did not result in increased phosphorus-uptake ( Figure 2 (b)). Furthermore, increased Phosphorus fertilization resulted in decreased growth in internode length, leaf area, petiole, vine length, nodes production, although this was not significant (P<0.05). This datum also showed a decline in fresh root biomass accumulation ( Figure 3 (f)) following increased phosphorus fertilization corresponding with Rashid & Waithaka (2009) findings.

The increased fresh root biomass concurs with Kareem (2013) report, that root growth particularly development of lateral roots and fibrous rootlets is encouraged by phosphorus. It is reported that phosphorus is one of the most important nutrients for many plant species including sweetpotato ( El Sayed Hameda *et al*., 2011). The maximum increase in node production, above ground fresh biomass and fresh root biomass was recorded at 60 ppm phosphorus fertilization ( Table 4). This indicates that 60 ppm was the approximate optimal concentration for sweetpotato vine growth.

### Calcium rate

**Figure 5.**
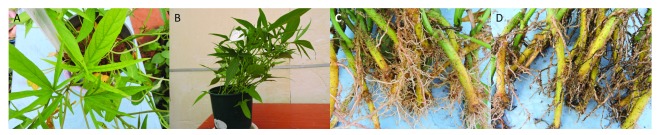
Ca deficient vines on the minus Ca treatment ( **A**), sweetpotato vines on all nutrient control ( **B**), ( **C**) sweetpotato roots on all nutrient control against Ca deficient sweetpotato roots on minus Ca treatment ( **D**) sampled 45 days from planting.

Increased calcium fertilization resulted in a significant ( Table 4) increase in the means of internode length, leaf area, vine girth and vine length ( Table 6). Treatments with calcium omitted resulted in vines with reduced internode length, leaf area, vine length, fewer nodes and low above ground fresh biomass accumulation, as well as low calcium concentrations in the leaf tissue. Chlorosis, curling, cupping and distortion of younger growing apical leaves was also observed ( Figure 5 (A)) against all nutrient control in which plants were vigorous and healthy ( Figure 5 (B)). Plants exhibited detrimental effects of advanced root rot in the calcium omitted nutrient media treatment 45 days from planting ( Figure 5 (D)), while plants in the all nutrient control exhibited no root growth anormalies ( Figure 5 (C)). 

**Table 6.** Effect of calcium fertilization on vegetative growth parameters of sweetpotato plants 45 days from planting.

Ca (ppm)	IL (cm)	LA (cm ^2^)	PL (cm)	VG (cm)	VL (cm)	Nodes	Ca leaf tissue (%)	AGFW (g)
0	1.7 ^b^	35.8 ^b^	9.3	0.9	27 ^b^	3.7	0.87	17
100	2.0 ^ba^	46.1 ^ba^	12.4	0.9	37 ^a^	4.5	1.56	27
200	2.4 ^a^	47.1 ^a^	11.8	0.9	36.4 ^a^	4.2	2.39	27.1
300	2.3 ^a^	44.8 ^ba^	11.9	0.9	33.5 ^a^	4.4	3.39	20.6
400	2.1 ^ba^	45.5 ^ba^	11.2	1.0	33.6 ^a^	4.2	3.52	25.5
LSD (5%)			ns	ns	ns			

*Means followed by the same letter within a column are not significantly different from one another; ns = not significant; IL = internode length; LA = leaf area; PL = petiole length; VG= vine girth; VL = vine length; Ca = calcium; AGFW = above ground fresh weight.*

Mild chlorosis and misshapen apical leaves were noted when plants were fertigated with 100 ppm calcium. The maximum significant ( Table 4) increase in leaf area, vine length and internode length were recorded at 200 ppm. Maximum accumulation in above ground fresh biomass accumulation was also observed at 200 ppm Ca fertigation ( Table 6). These results show that increased calcium fertilization results in increased growth of leaf area, plant height, petiole and internode length and fresh biomass accumulation as calcium accumulation in the leaf tissue increased up to 2.39%. Further increase in the calcium fertigation rate resulted in the decline in growth of the measurable parameters above ( Table 6). There was no significant difference in petiole length and nodes production because of increased calcium fertigation. The highest growth in the vine length was recorded at 100 ppm and 200 ppm which was 37 and 36.4 cm respectively, but not significantly different ( Table 6).

Chlorosis, curling and distortion of younger growing apical leaves in calcium omitted nutrient media corroborated with report by Crasswell *et al.*, 1995. Sweetpotato cultivars grown in the Pacific region on calcium deficient soils exhibited similar symptoms ( Crasswell *et al.* (1995). Generally, calcium deficient plants recorded significantly reduced growth (P<0.05) in internode length, leaf area and vine length ( Table 6). There was also decreased fresh weight biomass and limited calcium tissue accumulation ( Table 6 and Figure 2 (c) respectively), this is because the root tips of calcium deficient sweetpotato become rotten and fail to grow making it impossible for absorption of other nutrients to occur ( Bautista *et al.* (2018). In this study plants in the calcium omitted treatment further exhibited detrimental effects of advanced root rot 45 days after planting ( Figure 5 (D)) confirming the reports of O’Sullivan *et al.* (1997) and Ila’ava (1997). Similar results were also reported in taro ( *Colocasia esculenta*) Miyasaka *et al.* (2002). The pronounced detrimental effect of root die back is attributed to inadequate iron uptake resulting from poor root function. Further documented work by O’Sullivan *et al.* (1996a) reported that iron deficiency may be induced by calcium nutrition disorder resulting in adverse effects on roots.

The present data ( Table 6) additionally show a significant increase in internode length, leaf area and vine length with increased calcium fertigation up to 200 ppm, after which no further growth was recorded. Maximum growth in leaf area, internode length, vine length and above the ground fresh weight was also recorded at 200 ppm calcium. These results confirm documented work by Hassan *et al.* (2014).

The positive effect of calcium on the significant growth of sweetpotato vegetative internode length, leaf area, vine girth and vine length might have been due to the calcium functions as a second messenger in the signal conduction between environmental factors and plant responses in terms of growth and development ( Bothwell & Ng, 2005; Delian *et al*., 2014; Harper *et al*., 2004; Hetherington & Brownlee, 2004; Hirschi, 2004; Reddy, 2001; Reddy & Reddy, 2004). Moreover, calcium ions play a pivotal role in membrane stabilization and in the regulation of enzymes synthesis ( Schmitz-Eiberger *et al*., 2002).

The superior effects of calcium at 200 ppm in enhancing the growth parameters described above may also be due to its physiological effects on sweetpotato plants. The positive effects of calcium on vine yield and its components might be due to calcium being a component of the middle lamella and is essential for intracellular membrane transport. Likewise, calcium is known to act as a signaling molecule that regulates metabolism, controlling respiration and reducing ethylene production in plants ( Hassan *et al*., 2014; Marschner, 2013). Therefore, 200 ppm calcium can be approximated to be the optimal concentration for sweetpotato vine growth.

### Sulfur rate

**Figure 6.**
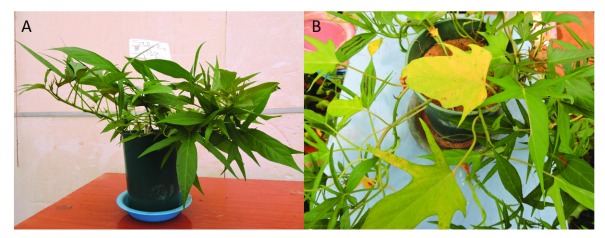
( **A**) Photograph of all nutrient control against the ( **B**) S deficient vines on the minus S treatment showing entire yellowing of the whole plant representative of S deficiency sampled 45 days after planting.

There was a non-significant decrease in growth in leaf area ( Table 7) and less accumulation of sulfur in leaf tissues in plants on S omitted nutrient media ( Figure 2 (d)). Yellowing of middle growing leaves was visualized one month after planting followed by entire yellowing of the whole plant on the minus S treatments ( Figure 6 (B) whereas healthy vigorous plants were observed on the all nutrient treatments ( Figure 6 (A)).

**Table 7.** The effect of sequentially increasing sulfur fertigation rate on vine, petiole and internode length, leaf area, vine girth, nodes production, fresh biomass accumulation and sulfur concentration in the leaf tissue.

S (ppm)	IL (cm)	LA (cm ^2^)	PL (cm)	VG (cm)	VL (cm)	Nodes	S leaf tissue (%)	AGFW (g)
0	2.2	39.6 ^b^	12.5 ^ba^	0.9	32.3	4.1	1.2	24.8
30	2.1	44.1 ^ba^	12.2 ^ba^	1.0	36.1	4.5	2.4	23.6
60	2.3	51.0 ^a^	13.2 ^ba^	0.9	35.5	4.4	3.1	23.9
90	2.1	45.7 ^ba^	11.3 ^b^	0.9	31.4	4.1	3.1	22.6
120	2.5	43.8 ^ba^	14.3 ^a^	0.9	35.5	4.3	3.4	23.0
LSD (5%)	ns			ns	ns	ns		

*Means followed by the same letter within a column are not significantly different from one another; ns = not significant; IL = internode length; LA = leaf area; PL = petiole length; VG = vine girth; VL = vine length; Nodes = number of 3 node cuttings per vine; S = sulfur; AGFW = above ground fresh weight.*

Increased fertilization with sulfur caused a significant increase in internode length at P≤0.05 ( Table 4). There were no significant differences in the means of vine length and vine girth because of increased sulfur fertilization. An increase in sulfur tissue accumulation was recorded following consecutive sulfur fertilization ( Figure 2 (d)) with the highest sulfur rate applied (120 ppm) corresponding to 0.34% sulfur tissue accumulation, which did not cause any significant growth of the measurable parameters. The highest growth in vine length were recorded at 30, 60 and 120 ppm S application which corresponded to 36.1, 35.5 and 35.5 cm respectively ( Figure 3 (c)), although not significantly different.

The increased linear non-statistical growth in leaf area, vine girth, petiole length, vine length and nodes production could be as result of sulfur acting as a signaling molecule involved in root formation, abiotic defense, and senescence ( Hu *et al*., 2012; Jin *et al*., 2011; Shan *et al*., 2014; Zhang *et al*., 2008; Zhang *et al.* 2009; Zhang *et al.* 2011). The sulfur application rates 0, 30, 60 and 90 ppm seemed to be lower based on the corresponding leaf tissue sulfur accumulation that was recovered in the leaf tissue as 0.12, 0.24, 0.31 and 0.31 ppm respectively and apparently did not have considerable significant effects on the linear increase of sweetpotato phenological growth parameters ( Figure 2 (d)), which conforms to the finding of Alu *et al.*, (2012). O’Sullivan *et al* (1997) quoted an approximate critical value of 0.34% extractable sulfur leaf tissue, below which sulfur deficiency is likely in sweetpotato, the latter findings conform with this study. A plot of the concentration of sulfur recovered in the 7 ^th^ to 9 ^th^ open leaf blades from the shoot tip as a percentage of each of the five treatment levels resulted in a luxury consumption within the plateau region ( Figure 2 (d)) of the curve relating sulfur tissue accumulation to increased sulfur fertilization. The concentrations of the index tissues remained approximately constant as the sulfur tissue neared 0.34% indicating the plant had been satisfied. Furthermore, no significant increase in phenological growth parameters was observed. Similar results were reported by O’Sullivan *et al.* (1997). These results indicated that 0.34% sulfur tissue occurred with 120 ppm sulfur fertilization, suggesting this is the approximate optimal concentration in sweetpotato vine growth. Thus, sulfur fertilization rates that had extractable sulfur content in the leaf tissues below 0.34% were identified as deficient.

### Boron rate

**Figure 7.**
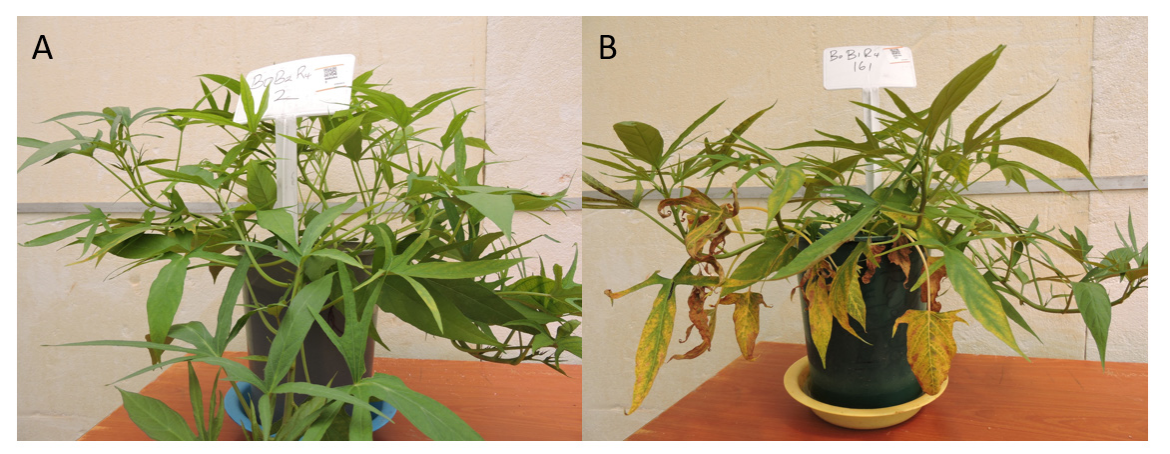
Early symptoms of boron deficiency ( **A**), severe symptoms of boron deficiency ( **B**).

Boron deficiency symptoms were observed 21 days after planting in the boron omitted nutrient media. Plants exhibited chlorosis in the apical leaves spreading to the basal foliage in the later stages of growth ( Figure 7 (A)). At advanced stage of vine growth, 45 days after planting, there was necrosis in the symptomatic leaves, death of the severely affected leaves and apical buds, eventually leading to premature plant senescence ( Figure 7 (B)). Boron treated plants with 0.1 ppm exhibited chlorosis of apical buds and necrosis in older growing leaves, but the crops life cycle was not terminated.

Successive increase in boron fertilization resulted in a significant increase ( Table 4) in growth of leaf area and vine length. The data also showed increased, but not significant (P<0.05), growth in petiole length, vine girth, and nodes production, however, the differences in the means of internode length was significant (P=0.05) between 0 ppm and 0.3 ppm Boron fertilized plants. Growth in vine internode, petiole length, vine girth and nodes produced decreased, but not significantly (P<0.05), under boron deficient treatments compared to the controls. Leaf area and vine length were significantly (P<0.05) reduced under Boron deficient treatments ( Table 8). Vine length declined from 36.2 to 27.7 cm and leaf area dropped by 9.4 cm ^2^ plant ^-1^ at 0 and 0.3 B application respectively indicating that boron had a significant role on the growth of sweetpotato vines. Increase in above the ground fresh biomass and boron tissue accumulation was also observed ( Table 8).

**Table 8.** The effect of successively increasing boron fertigation rate on vine, petiole and internode length, leaf area, vine girth, nodes production, above ground fresh biomass accumulation and boron concentration in the leaf tissue.

B (ppm)	IL (cm)	LA (cm ^2^)	PL (cm)	VG (cm)	VL (cm)	Nodes	B leaf tissue (%)	AGFW(g)
0	1.8 ^b^	40.4 ^b^	9.8	0.9	27.7 ^b^	3.9	0.00266	20.4
0.1	2.1 ^ba^	43.0 ^ba^	12.2	0.9	31.8 ^ba^	4.0	0.00573	23.8
0.2	2.3 ^a^	48.1 ^ba^	12.4	0.9	31.0 ^ba^	4.0	0.00710	25.8
0.3	2.2 ^ba^	49.8 ^a^	11.4	1.0	36.2 ^a^	4.3	0.00896	28.8
0.4	2.1 ^ba^	46.9 ^ba^	10.9	0.9	32.6 ^ba^	4.0	0.01230	25.6
LSD (5%)		ns	ns	ns		ns		

*Means followed by the same letter within a column are not significantly different from one another according to Dunnett test. IL = internode length; LA = leaf area; PL = petiole length; VG = vine girth; VL = vine length; Nodes = number of 3 node cuttings per vine; B = Boron; AGFW = above ground fresh weight.*

Tissue analysis of the 7 ^th^ to 9 ^th^ open leaf blades from the shoot tip showed that boron levels in the leaf tissue increased successively with increased boron fertilization ( Figure 2 (e)), and this resulted in the increased growth of leaf area, vine length, nodes production and fresh biomass accumulation ( Table 8). Maximum growth in vine length was recorded at 0.3 ppm B application ( Figure 3 (b)), after Boron tissue level exceeded 0.00896% as boron fertilization increased, growth increase in phenological measurable growth parameters declined.

Li *et al.* (2017) reported that boron not only affects formation and development of reproductive organs of plants, but also plays an essential role in the vegetative growth of plants and participates in the structural composition of cell walls and membranes. The observed premature senescence and plant growth cycle termination could have been due to root elongation inhibition that affected haulm growth and development ( Dell & Huang, 1997; Han *et al*., 2008; Miwa *et al*., 2007). An important reason is that the root and leaf are vital organs of plant to acquire nutrients ( Li *et al*., 2017).

Similar results were documented by preceding boron studies in other crops like cotton ( *Gossypium hirsutum* L.) which reported that a transient deficiency of boron can lead to irreversible damage thus seriously affecting yields ( Rosolem & Costa, 2000).

It is further reported that boron deficiency indirectly affects the metabolism of proteins and nucleic acids and mediates the levels of hormones and phenolic substances in the plant body ( Beato *et al*., 2010; Zhou *et al*., 2015). This confirms the advanced chlorosis and premature senescence of plants that was observed ( Figure 7 (B)). The percentage of boron concentration recovered in the 7 ^th^ to 9 ^th^ leaf blades increased linearly from 26.6 ppm to 123 ppm as the concentration of boron fertilization increased from 0 ppm to 0.4 ppm respectively ( Figure 2 €). Leaf tissue boron concentration plotted against vine yield and its components showed that maximum growth in leaf area, vine girth, vine length, nodes produced, and accumulation of fresh biomass was achieved at 0.3 ppm boron fertilization, corresponding to 89.6 ppm boron leaf tissue accumulation. O’Sullivan *et al.* (1997) reported a sufficiency range of 50 – 200 ppm measured in the 7 ^th^ to 9 ^th^ open leaf blades from the shoot tip. In this study fertilization of plants with 0.4 ppm resulted in B tissue accumulation of 123 ppm Boron culminating in decreased growth of vine internode, leaf area, petiole length, vine girth, vine length, nodes produced and fresh biomass ( Table 8). This data points out that in sweetpotato, when boron leaf tissue exceeds 89.6 ppm it becomes toxic to plant, indicating 0.3 ppm is the optimal boron fertilization in sweetpotato vine production.

## Conclusion and recommendations

The study demonstrated that nutrient media composition is very critical to the production of sweetpotato vines as pre-basic seed using the sandponics system. By sequentially increasing the application rate of nitrogen, phosphorus, calcium, sulfur and boron the variations observed in both the growth parameters and the accumulation of elements in the leaf tissue have been used to identify the optimal application rates for each element in the nutrient media for the sandponics system ( Table 9). Differences in growth parameters like vine internode length, leaf area, petiole length, vine girth, vine length, nodes produced, and fresh biomass were readily attributed to the application rate of nutrients. Although, this optimized nutrient media has been formulated using cultivar ‘Kabode’ it could easily be extended for multiplying other sweetpotato varieties with some slight modifications. We therefore recommend a follow up study to adapt the nutrient media for other sweetpotato varieties. The study also identified nutrient deficiency symptoms for various elements that can be used to guide sandponics operations in correcting for nutrient deficiency ( Table 9).

**Table 9.** Optimized nutrient media and nutrient deficiency symptoms for vine multiplication using the sandponics system.

Element	Optimal application rate (ppm)	Probable source	Nutrient deficiency symptoms
Nitrogen	200	Calcium nitrate (Ca(NO) _2_)	Stunted plants with minimal expansion of leaf area. Reddening of basal leaf edges advancing to younger growing leaves.
Phosphorus	60	Calcium triple phosphate (TSP)	Yellowing of older leaves spreading from discrete interveinal patches affecting half of the blades.
Calcium	200	Calcium nitrate (Ca(NO) _2_)	Chlorosis, curling, cupping and distortion of younger growing apical leaves. Plants exhibit detrimental effects of root rot.
Sulfur	120	Magnesium sulfate (MgSO4)	Yellowing of middle growing leaves succeeded with entire yellowing of the whole plant.
Boron	0.3	Microsol B	Chlorosis in the apical leaves spreading to the basal foliage in the later stages of growth. Necrosis in the symptomatic leaves at advanced stage of vine growth, death of severely affected leaves and apical buds eventually leading to premature plant senescence.

Sandponics is a promising technology for breaking the limited supply of pre-basic seed in sweetpotato cropping systems. It is vital to optimize critical factors to maximize vine production in sandponics. For instance, previously, ( Wanjala *et al*., 2018 pers comm) had indicated that despite 30% increase in vine multiplication rate in sandponics, vine production by the conventional method of using soil was still more cost effective. Therefore, we recommend a follow up study to compare the costs and benefits of the two systems using this optimized sandponics media.

## Data availability

Relationships between sweetpotato vegetative growth parameters and the supply of N, B, S, Ca and P across a range of levels is deposited at Open Science Framework.

OSF. Optimization of nutrient media for sweetpotato (Ipomoea batatas L.) vine multiplication in sandponics: Unlocking the adoption and utilization of improved varieties, https://doi.org/10.17605/OSF.IO/HK3T4. ( Makokha, 2018)

The dataset contains the following files:

Dataset. 001. Relationship between sweetpotato vegetative growth parameters and the supply of N P Ca S & B across a range of levels.

Dataset: 002. Composite fresh and dry above ground biomass of sweetpotato vines as affected by increased fertilization of N, P, Ca, S & B.

Dataset. 003. Composite fresh and dry root biomass of sweetpotato vines as affected by increased fertilization of N, P, Ca, S & B.

Data is available under a CC.0 1.0 Universal License
