# Supplementary material for: Optimization of nutrient media for sweetpotato ( Ipomoea batatas L.) vine multiplication in sandponics: Unlocking the adoption and utilization of improved varieties
Source: Gates Open Res. 2018 Nov 19;2:59. [Version 1] doi: 10.12688/gatesopenres.12879.1 (PMC6635673; doi:10.12688/gatesopenres.12879.1)
Supplement: Peer review history

# Peer review: Reviewer response for version 1

**DOI:** 10.21956/gatesopenres.13971.r27416

**Published:** 2019-07-15

**Authors:** Martin Chiona

**Affiliations:** 1 Mansa Research Station, Zambia Agriculture Research Institute (ZARI), Mansa, Zambia

Generally the paper is well written and scientifically sound in terms of design and conduct of the experiment. However, I have some suggested editorials for consideration.

 Page 4, under **Petiole length (cm) **second line: Consider adding “lamina” after the second “…leaf”

   

**Results and Discussion**

 Page 4, under Nitrogen rate, first paragraph: Consider replacing “on received all nutrient” with “that received all nutrients”

 Page 4, under Nitrogen rate, fourth paragraph, line 4: Change “N concentration” to “N application rate”

 Page 4, under Nitrogen rate, fourth paragraph, line 12: consider replacing “level” with “concentration”

 Page 4, under Nitrogen rate, fourth paragraph, line 13: Replace “when its value was relatively lower than” with “till”.

 Page 4, under Nitrogen rate, fourth paragraph, line 14: consider replacing “level” with “concentration”

 Page 4, under Nitrogen rate, fourth paragraph, line 16: Replace “increment of N levels in the plant tissue” with “advantage in increasing N levels.”

 Page 4, under Nitrogen rate, fourth paragraph, line 17: Replace “when N tissue was approximately 5.01% or above it” with “N application levels beyond 200 ppm”

 Page 4, under Nitrogen rate, fourth paragraph, line 20: Insert “levels” between “fertilization” and “increased”

 Page 4, under Nitrogen rate, fourth paragraph, line 20: Replace “until 5.01% N accumulation in plant tissue when” with “plant tissue N concentration to 5.01% beyond which”

 Page 4, under Nitrogen rate, fourth paragraph, line 22: Insert “in plant” between “N” and “tissue”

 Page 5, under Nitrogen rate, figure 2(f): Reverse the axis so that “N supplied (ppm)” becomes the x-axis so the picture can become clearer.

 Page 6, under Nitrogen rate, Table 3: This table can be fused into Table 4 by adding two columns to Table 4 to accommodate the two extra columns (N leaf level (%) and AGFW (g)) included in Table 3.

 Page 6, under Nitrogen rate, Table 3: For the 150 ppm level, the AGFW (g) is much higher than what was obtained for the much higher levels of 200 and 250 ppm N application. This needs to be explained.

 Page 6, line 3: Insert “below” between “approximately” and “4.0%”

 Page 6, under Boron leaf tissue accumulation as affected by increased nitrogen application, second paragraph, line 4: Replace “greenhouse studies” with “greenhouses”.

 Page 6, under Boron leaf tissue accumulation as affected by increased nitrogen application, third paragraph, line 3: Insert “accumulation in” between “nitrogen” and “tissue”.

 Page 6, under Boron leaf tissue accumulation as affected by increased nitrogen application, second paragraph, line 5: Replace “above 5.01% nitrogen tissue” with “leaf tissue nitrogen concentration of above 5.01%”.

 Page 6, under Boron leaf tissue accumulation as affected by increased nitrogen application, second paragraph, line 10: Insert “; respectively” before the citation.

 Page 6, under Phosphorus rate, first paragraph, line 4: Replace “growth” with “length”.

 Page 9, under Phosphorus rate, first paragraph, line 2: Insert “accumulation” before “reached”

 Page 9, under Phosphorus rate, first paragraph, line 5: Replace “leaf tissue” with “application levels”.

 Page 9, under Phosphorus rate, first paragraph, line 1-5: Check P leaf tissue % at 120 ppm, it went up contradicting your statement. Please clarify.

 Page 9, under Phosphorus rate, second paragraph, line 9: Delete “documented”.

 Page 9, under Phosphorus rate, third paragraph, line 17: Insert “beyond 60 ppm” before “resulted”

 Page 10, under Calcium rate, first paragraph, line 7: Delete “above”

 Page 11, under Sulfur rate, second paragraph, Line 1: The internode length was not consistently increasing with increased levels of the S applied. From your data (Table 4) S does not seem to be affecting internode length that much as the variation up and down attests. In some cases, no S had longer internodes than 30 and 60 ppm.

 Page 13, under Boron rate, fourth paragraph, line 1: Consider replacing “documented by preceding” with “reported by previous”

 The information in Table 4 is also repeated in Tables 3, 5, 6, 7 and 8. My suggestion is that you delete Table 4 and add the summary statistics (mean, LSD and CV) at the bottom of the respective tables.

 The subtitle “unlocking the adoption and utilization of improved varieties" does not seem to have been addressed. You may need to consider dropping it.

I confirm that I have read this submission and believe that I have an appropriate level of expertise to confirm that it is of an acceptable scientific standard.

# Peer review: Reviewer response for version 1

**DOI:** 10.21956/gatesopenres.13971.r26811

**Published:** 2018-12-19

**Authors:** Qiang Li, Yan Hui

**Affiliations:** 1 Xuzhou Institute of Agricultural Sciences in Jiangsu Xuhuai District, Key Laboratory for Biology and Genetic Breeding of Sweetpotato (Xuzhou), Ministry of Agriculture, Sweet Potato Research Institute (SPRI), Chinese Academy of Agricultural Sciences, Xuzhou, China; 2 Sweet Potato Research Institute (SPRI), Chinese Academy of Agricultural Sciences, Xuzhou, China

The study is designed appropriately and the work is technically sound, but somethings need to be refined.

1. To better design the experiment and optimize the nutrient media, the composition of the sand soil should be assessed.
2. Why choose the trace elements of Boron? The fertilizer composition used in the experiment is 2.5% B and 0.036% Mo. We know Mo is also an important trace element in leaf growth ^1^, how to prove the symptoms of yellow leaves was caused by B deficiency?
3. Duplicate content reduction. The content of tables and figures needs to be simplified, for example, we can obtain the information from Figure 3 in Table 4.

We confirm that we have read this submission and believe that we have an appropriate level of expertise to confirm that it is of an acceptable scientific standard.

## References

[1] : Effects of selected trace elements on plant growth. *Journal of the Science of Food and Agriculture*.1990;51(4) : 10.1002/jsfa.2740510404 447-479 10.1002/jsfa.2740510404
